# Cobalt protoporphyrin IX induces transient, dose- and time-dependent granulocyte mobilization with mild metabolic effects in mice

**DOI:** 10.1007/s43440-025-00751-4

**Published:** 2025-07-09

**Authors:** Aleksandra Bednarz, Paweł Kożuch, Kacper Kowalski, Izabella Skulimowska, Neli Kachamakova-Trojanowska, Jadwiga Filipek-Gorzała, Patrycja Kwiecińska, Raquel García-García, Kinga Gawlińska, Kinga Mależyna, Andrzej Kubiak, Natalia Bryniarska-Kubiak, Alicja Józkowicz, Krzysztof Szade, Agata Szade

**Affiliations:** 1 Department of Medical Biotechnology, Faculty of Biochemistry, Biophysics and Biotechnology, Jagiellonian University, ul. Gronostajowa 7, Kraków, 30-387 Poland; 2 Laboratory of Stem Cell Biology, Faculty of Biochemistry, Biophysics and Biotechnology, Jagiellonian University, Kraków, Poland; 3 Malopolska Centre of Biotechnology, Jagiellonian University, Kraków, Poland; 4 Doctoral School of Exact and Natural Sciences, Jagiellonian University, Kraków, Poland

**Keywords:** CoPPIX, Hmox1, Liver markers, Kidney markers, Filgrastim

## Abstract

**Background:**

Recombinant granulocyte colony-stimulating factor (G-CSF) is the most commonly used agent for treating neutropenia and mobilizing hematopoietic stem cells (HSCs) for transplantation. However, some patients do not respond effectively to the currently used mobilization protocols. To address this, new therapeutic approaches are needed. A potential strategy is pharmacological induction of endogenous mobilizing factors via cobalt protoporphyrin IX (CoPP). CoPP mobilizes HSCs and granulocytes by increasing endogenous G-CSF, though optimal dosing and potential side effects remain unclear. Our study aimed to optimize CoPP dosing and timing, and assess its safety in mobilizing cells from bone marrow to blood.

**Methods:**

Mice were treated with different doses of CoPP, and blood cell counts, cytokine concentrations, and organ damage markers were evaluated at various time points after injection.

**Results:**

Our results show that CoPP exerts a dose-dependent mobilizing effect, with the highest G-CSF levels and number of mobilized leukocytes observed in mice treated with 10 mg/kg of CoPP. While there were no severe adverse effects, there were mild fluctuations in markers of organ function, including a reduction in blood urea nitrogen (BUN) and glucose levels during the five days of administration. Additionally, although most parameters normalized within 30 days, the decrease in BUN persisted. Mice experienced short-term weight loss following CoPP administration, but they regained their initial weight within two weeks.

**Conclusions:**

This study demonstrates that CoPP mobilizes cells from the bone marrow to the blood in a dose-dependent manner, with mild side effects, including temporary changes in biochemical markers and a sustained reduction in BUN levels.

**Supplementary Information:**

The online version contains supplementary material available at 10.1007/s43440-025-00751-4.

## Introduction

Cobalt protoporphyrin IX (CoPP) is a compound that has been widely used to induce the expression of heme oxygenase-1 (HO-1 or Hmox1) since the 1970s [1]. HO-1 is an enzyme that degrades heme into ferrous ions (Fe²⁺), carbon monoxide (CO), and biliverdin, which is subsequently converted to bilirubin by biliverdin reductase [2]. The products of HO-1 activity exert various effects, including immunomodulatory [3–5], antiapoptotic [6], and antioxidant [7] functions.

Although CoPP is commonly used as a model activator of HO-1 expression, several HO-1-independent CoPP activities have been identified. These include the inhibition of caspase-3 and − 8 activity [8], suppression of lipopolysaccharide (LPS)-induced activation of inducible nitric oxide synthase in RAW264.7 macrophages [9], and upregulation of cyclooxygenase-2 [10].

We demonstrated that CoPP can mobilize cells from the bone marrow to the blood independently of HO-1 activity [11]. Mobilization refers to the accelerated release of hematopoietic cells from their niche in the bone marrow into peripheral blood [12, 13]. This process can occur naturally, for example, during infection, but can also be induced pharmacologically. Pharmacological mobilization is primarily used to treat neutropenia (an abnormally low number of granulocytes in the blood) [14] or to collect hematopoietic stem cells (HSCs) for transplantation [15, 16]. The most commonly used mobilizing agent is recombinant granulocyte colony-stimulating factor (G-CSF) [17]. However, as not all patients respond effectively to currently available drugs [18], new therapeutic approaches are being explored, including the induction of endogenous mobilizing cytokines [19].

Mobilization with CoPP offers several advantages over the use of recombinant G-CSF. First, CoPP mobilizes a higher number of hematopoietic stem and progenitor cells (HSPCs) than recombinant G-CSF [11], which is critical for the success of hematopoietic cell transplantation [20]. Second, granulocytes mobilized by CoPP exhibit a more mature phenotype, characterized by increased granularity and higher expression of the Ly6G marker, compared to those mobilized by G-CSF [11]. This is particularly significant for treating neutropenia caused by anticancer therapy, as granulocytes mobilized by G-CSF tend to be less mature and are impaired in their ability to combat certain pathogens, such as *Candida albicans* [21]. Finally, CoPP mobilizes fewer T cells than G-CSF [11]. Since T cells are the primary drivers of graft-versus-host disease, this observation might have implications for reducing the risk of this serious complication in hematopoietic cell transplantation [22]. However, further studies are needed to confirm this potential benefit.

Given those advantages of CoPP in mobilizing cells from the bone marrow to the blood, we sought to evaluate its potential as a pharmacological mobilizing agent in a mouse model. Specifically, we aimed to determine the minimal effective dose and treatment duration to reduce potential side effects. Previous study by Muhoberac et al. [23] reported the toxic effects of CoPP, evaluating its use as a model for cytochrome P450-centered hepatic dysfunction. The study demonstrated that CoPP causes a dose-dependent depletion of liver cytochrome P450 in rats. This finding has significant clinical implications, as cytochrome P450 enzymes play a crucial role in the metabolism of various endogenous and exogenous compounds. Disruption of these enzymes may result in altered therapeutic efficacy or increased toxicity [24, 25]. Additionally, CoPP was shown to reduce the activities of NADPH-cytochrome P450 reductase and NADH-cytochrome b5 [23].

Thus, we also monitored toxicity markers during and after successful mobilization. Additionally, we assessed the long-term effects of CoPP on hematopoiesis and the function of major organs. Since CoPP has been proposed as a potential treatment for ischemic diseases [26] or diabetic wounds [27], it is crucial to fully understand its impact on the hematopoietic system and establish a treatment regimen that does not adversely affect hematopoiesis.

## Materials and methods

### Mice

Animal work was done in accordance with the Guide for the Care and Use of Laboratory Animals (Directive 2010/63/EU of the European Parliament) and approved by the Second Local Ethical Committee for Animal Research in Kraków (approval numbers: 323/2020 and 252/2021). All experiments were performed on male C57BL/6J mice, which were bred and maintained at the Animal Facility of the Faculty of Biochemistry, Biophysics and Biotechnology. Experiments were performed in specific pathogen-free (SPF) conditions, with constant light/dark cycle (14/10 h) and continuous temperature and humidity monitoring. Mice were kept in groups of ≤ 5 in individually ventilated cages with appropriate housing conditions, environmental enrichment (e.g., chew toys, nesting material), and food and water ad libitum. To minimize stress and ensure acclimatization, each mouse underwent gentle handling prior to the start of the experimental procedures. Throughout the experimental period, animals were under continuous observation. In the event of any visible health changes, the issue was first discussed with animal facility personnel, and if necessary, a veterinarian was consulted, and appropriate interventions were implemented immediately. Animals in each cage were randomly assigned to all experimental groups.

### Mobilization experiments

Recombinant human G-CSF (Neupogen, Amgen) was administered at 250 µg/kg body weight (b.w.). Cobalt protoporphyrin (CoPP; Frontier Scientific, Cat# Co654-9) was dissolved in dimethyl sulfoxide (DMSO, Sigma-Aldrich, Cat# D8418-100ML) at 200 mg/ml and diluted 1:115 in 0.9% NaCl prior to injection. Mice were treated with CoPP at 10 mg/kg b.w. (15 µmol/kg), unless otherwise specified. Lower doses of 5 mg/kg and 1 mg/kg were also tested to assess potential dose-dependent effects, based on prior studies. A vehicle control consisting of DMSO diluted 1:115 in 0.9% NaCl was used. Compounds were administered *ip* according to the schedules described for each experiment. A 5-day treatment regimen was used in most experiments, reflecting clinical protocols for G-CSF in neutropenia and HSCs mobilization [28]. Since CoPP induces endogenous G-CSF production, this regimen was adopted for consistency. To assess the impact of treatment duration, mice received daily injections of CoPP, G-CSF, or vehicle for 1 to 5 consecutive days. Animals from each treatment group were sacrificed after the indicated treatment time (1, 2, 3, 4, or 5 days), and blood samples were collected. The 5-day group served as a reference, consistent with our established protocol. To assess the long-term effects of CoPP treatment, a 30-day follow-up was included, aligning with clinical monitoring periods for HSC donors post-donation. Unless otherwise noted, mice were euthanized using CO₂ 6 h after the final injection. Peripheral blood (PB) was collected from the vena cava using heparinized syringes. Complete blood counts were performed using a Vet abc Plus + analyzer (Horiba). PB was centrifuged at 600 × g for 10 min at 4 °C; plasma was stored at − 80 °C for further analysis, and the cell pellet was processed for flow cytometry. Each experimental group included five mice. Long-term CoPP effects were evaluated in two independent experiments.

### Flow cytometry

Bone marrow (BM) cells were isolated from femurs and tibias by mechanically crushing the bones in cold PBS with calcium and magnesium, supplemented with 2% fetal bovine serum, FBS and DNase I (Roche Cat# 11284932001, 0.025 mg/ml). The resulting suspension was filtered through a 100 μm strainer and centrifuged. After centrifugation, cell pellets from both BM and PB were depleted of red blood cells (RBCs) using a hypotonic lysis buffer and washed in FACS buffer (PBS without calcium and magnesium, supplemented with 2% FBS). Cells were resuspended in FACS buffer and stained with fluorochrome-conjugated antibodies for 30 min at 4 °C. Following staining, cells were washed and resuspended in FACS buffer for flow cytometric analysis. Samples were collected using an LSR Fortessa cytometer (BD) and analyzed using FlowJo software (BD). The following flow cytometry reagents were used: 7AAD (BD Biosciences Cat# 559925), BV786 rat anti-mouse CD45 (clone 30-F11, BD Biosciences Cat# 564225, RRID: AB_2716861), BV421 rat anti-mouse CD11b (clone M1/70, BD Biosciences Cat# 562605, RRID: AB_11152949), PerCP-Cy5.5 rat anti-mouse Ly6C (clone AL-21, BD Biosciences Cat# 560525, RRID: AB_1727558), BV650 rat anti-mouse Ly6G (clone 1A8, BD Biosciences Cat# 740554, RRID: AB_2740255), Alexa Fluor 700 rat anti-mouse CD45R (B220, clone RA3-6B2, BD Biosciences Cat# 557957, RRID: AB_396957), Alexa Fluor 647 rat anti-mouse CD3 Molecular Complex (clone 17A2, BD Biosciences Cat# 557869, RRID: AB_396912), PE-CF594 rat anti-mouse CD4 (clone RM4-5, BD Biosciences Cat# 562285, RRID: AB_11154410), FITC rat anti-mouse CD8a (clone 53 − 6.7, BD Biosciences Cat# 553031, RRID: AB_394568), PE rat anti-mouse CD25 (clone 3C7, BD Biosciences Cat# 553075, RRID: AB_394605), APC-eFluor780 rat anti-mouse CD117 (c-Kit, clone 2B8, eBioscience, Cat# 47-1171-82, RRID: AB_1272177), PE-Cy7 rat anti-mouse Ly6 A/E (Sca-1, clone D7, BD Biosciences Cat# 561021 RRID: AB_2034021), Alexa Fluor 700 anti-mouse Lineage Cocktail (anti-mouse CD3, clone 17A2; anti-mouse Ly6G/Ly6C, clone RB6-8C5; anti-mouse CD11b, clone M1/70; anti-mouse CD45R/B220, clone RA3-6B2; anti-mouse TER-119, clone Ter-119, Biolegend, Cat# 133313, RRID: AB_2715571). Gating strategies are provided in the Supplementary Materials (Suppl. Figure 1 and Suppl. Figure 2).

### Clinical biochemistry tests and cytokine concentration analysis

Organ toxicity markers were measured in previously frozen plasma samples using SpotChem EZ Chemistry Analyzer according to the manufacturer’s instructions, using test strips: Spotchem II Panel-V2 (Arkray, Cat# 102725) and Spotchem Stat1 (Arkray, Cat# 77183). Cytokine levels were measured in plasma samples using custom-designed Luminex multiplex assay kits (R&D Systems, ThermoFisher Scientific). Plasma samples were thawed at 4 °C, vortexed, and centrifuged at 16,000 × g for 4 min before use. The assay was performed following the manufacturer’s protocols. Briefly, samples and standards were incubated with antibody-conjugated magnetic beads, followed by sequential incubations with biotinylated detection antibodies and streptavidin-PE. Wash steps were performed manually using a magnetic plate. Plates were read on a Luminex 200 system (Millipore), and cytokine concentrations were calculated from standard curves using xPONENT software.

### Statistical analysis

Statistical analyses were performed using GraphPad Prism software (version 6.07). Data are presented as mean ± SEM or as mean with individual data points, as indicated in figure legends. For comparisons involving three or more groups, one-way ANOVA was applied, followed by appropriate post hoc tests (Sidak’s or Dunnett’s) depending on the experimental design. For experiments involving two independent variables – treatment group and time, two-way ANOVA was used to assess the main effects of each factor (group and time) and their interaction. The group factor refers to experimental conditions (e.g., control, G-CSF, CoPP), while the time factor refers to the time points at which measurements were taken. The interaction term tests whether the effect of one factor depends on the level of the other (e.g., whether treatment effects differ over time). When significant main or interaction effects were detected, post hoc comparisons were performed using Tukey’s test post hoc. In the main text, ANOVA results are reported with the F-statistic, including degrees of freedom (between-groups and within-group, respectively) and the corresponding p-value. Adjusted p-values obtained in post hoc comparisons are shown in the graphs. Grubbs’ test was used to identify and exclude significant outliers. A p-value ≤ 0.05 was considered statistically significant.

## Results

### CoPP-induced mobilization is dose-dependent

**Fig. 1:**
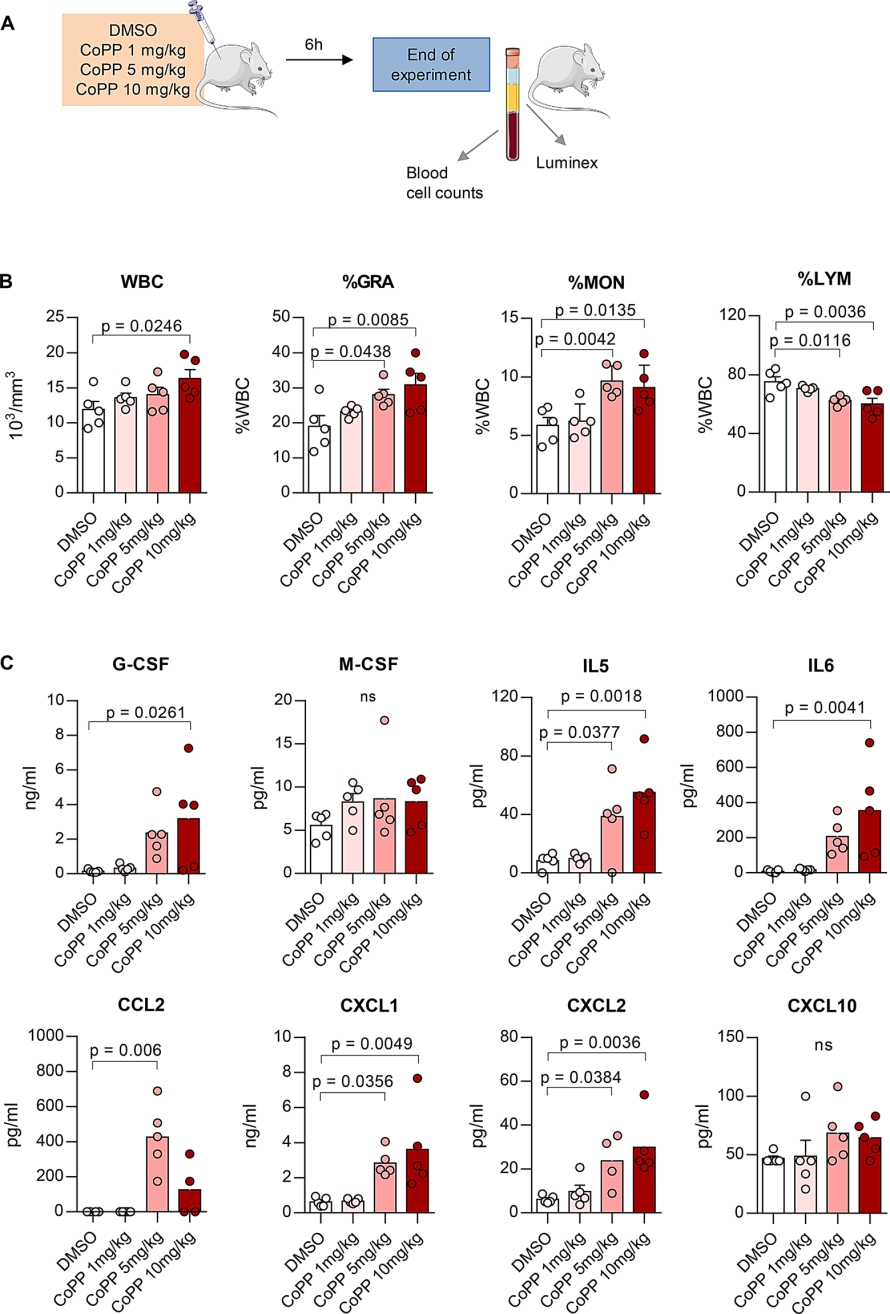
Cobalt protoporphyrin IX (CoPP) increases cytokine concentrations and induces granulocyte mobilization from the bone marrow to the blood in a dose-dependent manner. **A** Experimental scheme. C57BL/6J mice were injected once with CoPP or a solvent control (dimethyl sulfoxide, DMSO). Samples were collected 6 h after the injection. **B** Total leukocyte count in blood. CoPP dose-dependently increases the number of white blood cells (WBC). The percentages of granulocytes (%GRA) and monocytes (%MON) increase, while the percentage of lymphocytes (%LYM) decreases with increasing CoPP doses. **C** Selected cytokine and growth factor concentrations in plasma, measured by Luminex assay. CoPP dose-dependently increases the concentrations of granulocyte colony-stimulating factor (G-CSF), interleukin 5 (IL‐5), IL‐6, C-C Motif Chemokine Ligand 2 (CCL2 /MCP‐1), C-X-C Motif Chemokine Ligand 1 (CXCL1), and CXCL2. Results are shown as mean ± SEM. Statistical differences were assessed using one-way ANOVA followed by Dunnett’s post-hoc test. p-values from post-hoc tests are shown in the graphs. *n* = 5 mice per group

In our previous work, we demonstrated that a daily intraperitoneal injection of 10 mg/kg of CoPP for five days effectively mobilized cells from the bone marrow to the blood. However, in animal studies, CoPP doses range from as low as 1 mg/kg [29] to as high as 20 mg/kg [30]. As higher doses of CoPP can lead to toxicity, it is essential to explore safer dosing regimens. Therefore, we aimed to determine whether efficient mobilization could be achieved with lower doses of CoPP. We compared our standard 10 mg/kg dose of CoPP with lower doses (5 mg/kg and 1 mg/kg) in mice treated once with CoPP or a DMSO solvent control (Fig. 1A).

One-way ANOVA revealed a trend toward a dose-dependent effect of CoPP on white blood cell (WBC) counts (F₃,₁₆ = 2.955, *p* = 0.064, Fig. 1B), and the highest WBC count was observed in the 10 mg/kg group. There were significant differences in the percentages of granulocytes (F_3,16_ = 4.724, *p* = 0.0152, Fig. 1B), monocytes (F₃,₁₆ = 7.61, *p* = 0.0022, Fig. 1B) and lymphocytes (F₃,₁₆ = 6.632, *p* = 0.040, Fig. 1B). In DMSO-treated mice, granulocytes comprised 19.02 ± 3.08% of WBCs, whereas with increasing doses of CoPP, the proportion of granulocytes rose to 23.22 ± 0.67% (1 mg/kg), 28.1 ± 1.56% (5 mg/kg), and 30.8 ± 3.3% (10 mg/kg), with two higher doses (5 and 10 mg/kg) significantly increased in post hoc comparison. One-way ANOVA revealed a significant effect of CoPP dose on monocyte percentages. Post hoc comparisons indicated that the two higher doses (5 and 10 mg/kg) significantly increased monocyte percentages compared to the control (Fig. 1B). Conversely, the percentage of lymphocytes decreased with increasing doses of CoPP, from 75.12 ± 3.5% in DMSO-treated mice to 60 ± 3.89% in the 10 mg/kg group, with both the 5 and 10 mg/kg doses showing significant decreases in post hoc comparisons (Fig. 1B).

To gain a more detailed understanding of the immune cell populations affected in the blood, we performed flow cytometry analysis. CoPP treatment led to an increase in the number of mature neutrophils, identified by high expression of the Ly6G marker and increased granularity (Suppl. Figure 1 and Suppl. Figure 3). These cells are typically associated with acute inflammatory responses and microbial defense. In contrast, the number of eosinophils—another granulocyte subtype involved in allergic responses and parasitic infections—remained unchanged, as did the subpopulation of inflammatory monocytes characterized by high Ly6C expression (Suppl. Figure 3). Consistent with the complete blood count results, we observed no significant changes in the numbers of B cells or various subpopulations of CD4⁺ helper and CD8⁺ cytotoxic T cells (Suppl. Figure 3).

Given that G-CSF is the key mediator of CoPP-induced mobilization, we assessed its levels in plasma samples. One-way ANOVA revealed a significant effect of CoPP treatment on endogenous G-CSF levels (F₃,₁₆ = 4.185, *p* = 0.0229, Fig. 1C). Post hoc comparisons indicated that only the highest dose (10 mg/kg) significantly increased G-CSF concentrations compared to the DMSO-treated group. However, numerical increases were also observed at the lower doses: 1 mg/kg led to a 2.2-fold increase, and 5 mg/kg to a 16.3-fold increase. Similarly, interleukin 6 (IL-6) levels were significantly affected by CoPP treatment (F₃,₁₆ = 6.714, *p* = 0.038). Post hoc analysis showed that only the 10 mg/kg dose resulted in a statistically significant increase, with a 44-fold rise compared to the DMSO-treated group (Fig. 1C). One-way ANOVA revealed a significant effect of treatment on the levels of several mobilizing cytokines, including IL-5 (F₃,₁₆ = 8.487, *p* = 0.0013), C-X-C Motif Chemokine Ligand 1 (CXCL1, F₃,₁₆ = 7.240, *p* = 0.0028), CXCL2 (F₃,₁₅ = 6.838, *p* = 0.0040), and C-C Motif Chemokine Ligand 2 (CCL2/MCP-1, F₃,₁₄ = 12.16, *p* = 0.0003). Post hoc comparisons indicated that only the two higher doses (5 and 10 mg/kg) significantly elevated the concentrations of these cytokines compared to the control and lowest dose groups (Fig. 1C).

In summary, we found that CoPP-induced mobilization of cells from the bone marrow to the blood is dose-dependent. The minimal effective dose for achieving efficient mobilization in mice appears to be between 5 and 10 mg/kg.

### CoPP does not induce acute toxicity during a five-day treatment

**Fig. 2:**
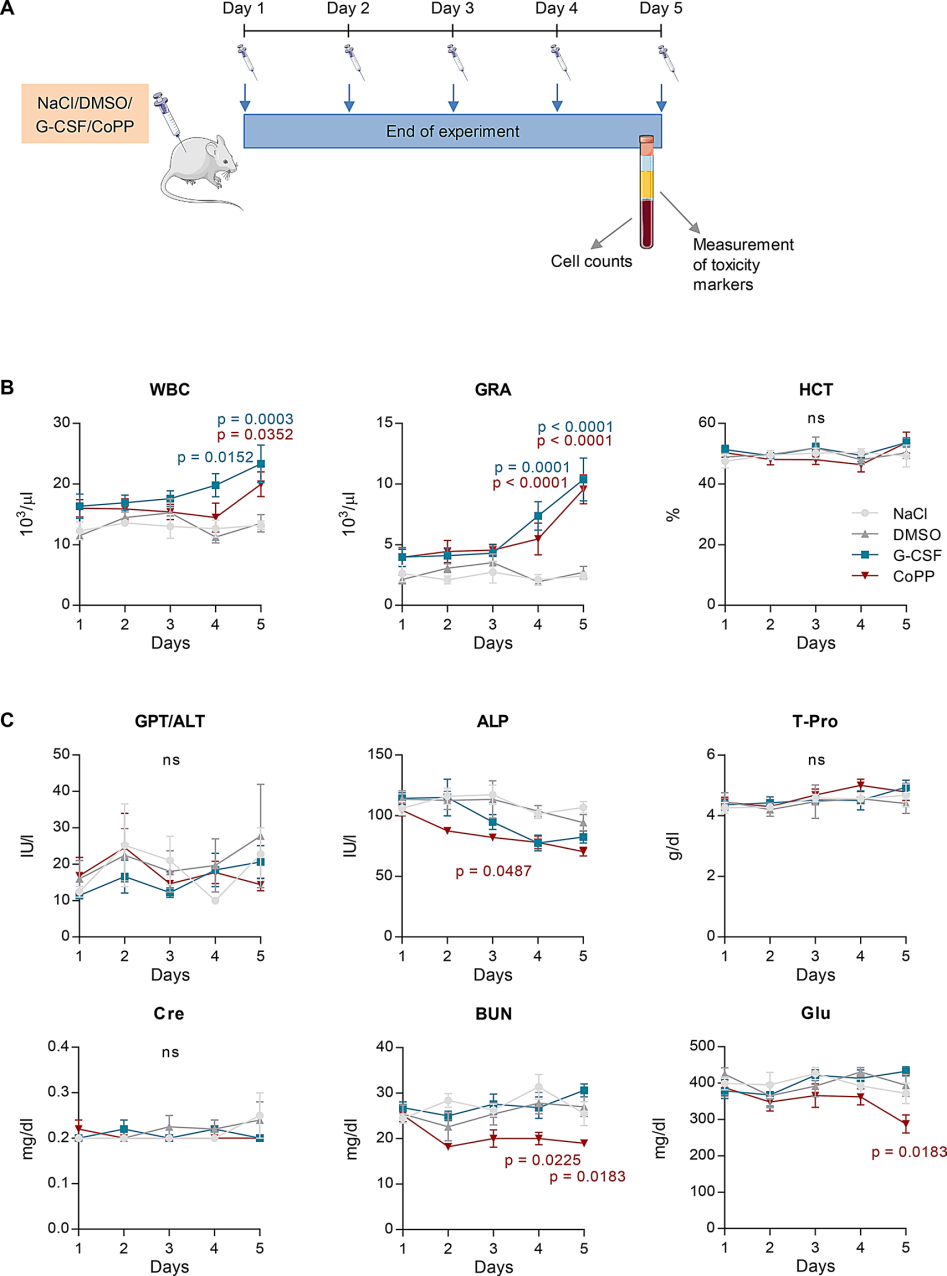
Cobalt protoporphyrin IX (CoPP)-induced mobilization of granulocytes increases with treatment duration and is associated with a decrease in plasma metabolic markers. **A** Experimental scheme. C57BL/6J mice were injected daily with 10 mg/kg CoPP, 250 µg/kg recombinant granulocyte colony-stimulating factor (G-CSF), or solvent controls (NaCl and dimethyl sulfoxide, DMSO) for 1 to 5 days. Samples were collected 6 h after the final injection. **B** Total leukocyte count in blood. Both recombinant G-CSF and CoPP increase the number of WBCs over 5 days of treatment. Granulocyte counts increase on days 1 to 3, with the greatest increases observed on days 4 and 5. A 5-day treatment with G-CSF or CoPP does not affect red blood cell parameters such as hematocrit (HCT). **C** Plasma organ function biomarkers measured using the SpotChem EZ Chemistry Analyzer. Treatment with G-CSF or CoPP does not affect alanine transaminase (ALT/GLP), total protein (T-Pro), or creatinine (Cre) levels. Both G-CSF and CoPP reduce plasma alkaline phosphatase (ALP) activity, but only CoPP decreases blood urea nitrogen (BUN) and glucose concentrations. Results are shown as mean ± SEM. Statistical differences were assessed using two-way ANOVA followed by Tukey’s post-hoc test. p-values from post-hoc tests comparing CoPP or G-CSF to their respective controls (DMSO or NaCl) are shown in the graphs. *n* = 5 mice per group

The standard protocol for G-CSF-induced mobilization of granulocytes, commonly used to prevent and/or treat neutropenia associated with cancer treatment, typically requires five days of administration. In our previous studies, we followed this standard protocol to evaluate the mobilizing potential of CoPP. However, we sought to determine whether a shorter treatment duration could still achieve sufficient granulocyte mobilization. To investigate this, we conducted a time-course experiment, treating mice with CoPP, recombinant G-CSF, or control solvents daily for one to five days (Fig. 2A). We observed the highest numbers of total leukocytes and granulocytes on day 5 in both CoPP- and G-CSF-treated groups. Two-way ANOVA showed that treatment group significantly affected WBC counts (F₃,₈₀ = 14.20, *p* < 0.0001), while the effect of time was modest (F₄,₈₀ = 2.604, *p* = 0.0420), and the interaction between time and treatment was not significant (F₁₂,₈₀ = 1.090, *p* = 0.3796, Fig. 2B). For granulocytes, we found significant effects of treatment group (F₃,₈₀ = 26.95, *p* < 0.0001), time (F₄,₈₀ = 9.138, *p* < 0.0001), and their interaction (F₁₂,₈₀ = 3.729, *p* = 0.0002), indicating that granulocyte levels increased over time in a treatment-dependent manner (Fig. 2B). In contrast, hematocrit (HCT) values remained stable. We did not detect significant effects of treatment (F₃,₈₀ = 0.9167, *p* = 0.4367), time (F₄,₈₀ = 1.492, *p* = 0.2123), or their interaction (F₁₂,₈₀ = 0.7027, *p* = 0.7444), suggesting that mobilization treatments did not affect erythrocyte parameters (Fig. 2B).

Previous studies have reported possible liver toxicity associated with CoPP [23]. To assess the safety of CoPP treatment in inducing mobilization, we measured biomarkers of organ function. Over the five-day treatment period, neither CoPP nor G-CSF affected alanine transaminase (GPT/ALT), creatinine, or total protein concentrations (Fig. 2C). However, both treatments slightly reduced alkaline phosphatase (ALP) activity. We observed the lowest ALP levels on day 4 in G-CSF-treated mice and on day 5 in CoPP-treated mice. Two-way ANOVA confirmed significant effects of treatment group (F₃,₇₈ = 12.95, *p* < 0.0001) and time (F₄,₇₈ = 7.531, *p* < 0.0001), but not their interaction (F₁₂,₇₈ = 1.303, *p* = 0.2339, Fig. 2C). In CoPP-treated mice, blood urea nitrogen (BUN) levels began to decline on day 2 and remained lower through day 5. We detected a significant effect of treatment group on BUN (F₃,₇₈ = 14.45, *p* < 0.0001), while time and interaction effects were not significant (F_4,78_ = 1.330, *p* = 0.2663 and F_12,78_ = 1.731, *p* = 0.0760, respectively, Fig. 2C). Glucose levels also decreased, most notably on day 5 (Fig. 2C). Here, we found a significant effect of type of treatment (F₃,₇₈ = 5.339, *p* = 0.0021), but no significant effects of time (F₄,₇₈ = 1.702, *p* = 0.1579) or interaction (F₁₂,₇₈ = 1.272, *p* = 0.2523).

In summary, efficient mobilization with CoPP requires five days of continuous treatment. Importantly, this five-day CoPP regimen does not significantly induce markers of acute stress but does reduce BUN and glucose levels.

### Most of the CoPP-affected parameters go back to normal levels by day 30

**Fig. 3:**
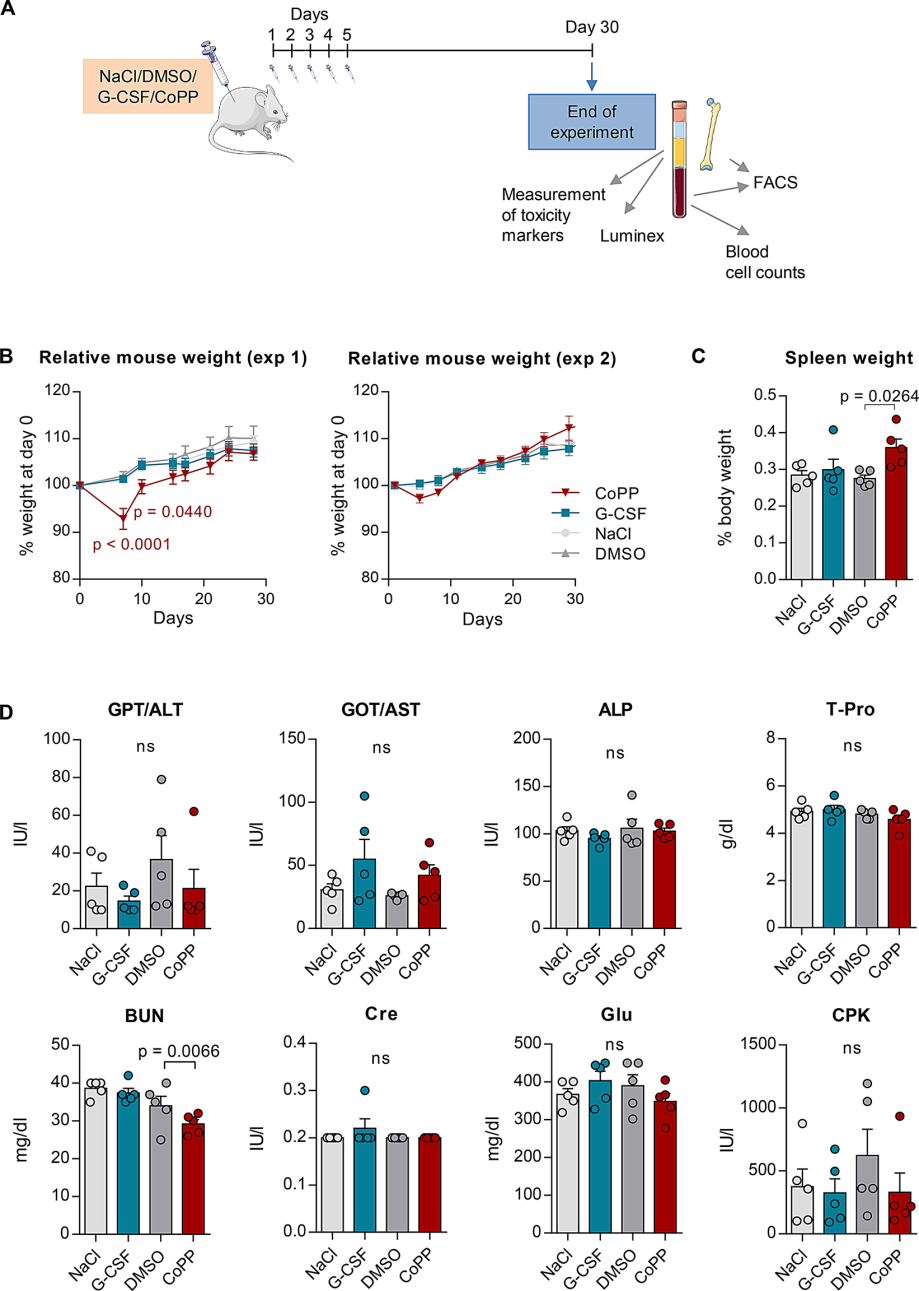
Most cobalt protoporphyrin IX (CoPP)-induced changes resolve within 25 days after stopping the mobilization treatment. **A** Experimental scheme. C57BL/6J mice were injected daily with 10 mg/kg CoPP, 250 µg/kg recombinant granulocyte colony-stimulating factor (G-CSF), or solvent controls (NaCl and dimethyl sulfoxide, DMSO) for 5 days. Samples were collected 25 days after the final injection. The experiment was repeated twice. **B** Relative mouse weight (data shown separately for the two independent experiments). CoPP causes a transient decrease in weight, with mice returning to their initial weight 5 days after the end of treatment. **C** Relative spleen weight. Mice treated with CoPP show an increase in spleen weight 25 days after treatment. **D** Organ function biomarkers in plasma, measured using the SpotChem EZ Chemistry Analyzer. Most liver and kidney function markers are similar between mobilized and non-mobilized mice 25 days after treatment, except for blood urea nitrogen (BUN), which remains significantly lower in CoPP-treated mice (GLP/ALT – alanine transaminase, GOT/AST – aspartate aminotransferase, ALP – alkaline phosphatase, T-Pro – total protein, Cre – creatinine, BUN – blood urea nitrogen, glucose, CPK – creatine phosphokinase). Results are shown as mean ± SEM. Statistical differences were assessed using two-way ANOVA with Tukey’s post-hoc test (B) and one-way ANOVA with Sidak’s post-hoc test (**C**–**D**). p-values from post-hoc tests are shown in the graphs. *n* = 5 mice per group

Next, we aimed to evaluate the long-term effects of CoPP-induced mobilization on both the hematopoietic system and overall homeostasis in mice. Mice were treated with CoPP or G-CSF for five days, and then followed for an additional 25 days (Fig. 3A). In line with previous studies, we observed a temporary decrease in body weight in CoPP-treated mice (Fig. 3B). The most pronounced reduction occurred on day 7 in the first experiment (7.1 ± 2.2% of initial weight) and on day 5 in the second experiment (2.88 ± 0.9% of initial weight). In Experiment 1, the interaction between time and group was not significant (F₂₁,₁₂₈ = 0.96, *p* = 0.52), but both time (F₇,₁₂₈ = 24.96, *p* < 0.0001) and group (F₃,₁₂₈ = 12.90, *p* < 0.0001) had significant effects. In Experiment 2, the interaction was also not significant (F₂₇,₁₆₀ = 1.10, *p* = 0.34), time had a significant effect (F₉,₁₆₀ = 55.20, *p* < 0.0001), and group did not (F₃,₁₆₀ = 0.65, *p* = 0.59). This weight loss was transient, and the mice regained their initial weight within five days after treatment ended, matching the weight of mice in other groups over the subsequent two weeks (Fig. 3B).

It is well-established that G-CSF treatment increases spleen weight during mobilization, and we found that CoPP had a similar effect [11]. To assess whether the spleen returned to its normal size after treatment, we measured spleen weight 25 days after CoPP treatment was terminated. We found that spleen weight remained elevated in CoPP-treated mice compared to DMSO-treated controls, while G-CSF-treated mice showed no such prolonged effect (F_3,16_ = 3.593, *p* = 0.0370, Fig. 3C).

At the end of the experiment, plasma samples were analyzed for markers of organ damage. One-way ANOVA revealed a significant effect of treatment on blood urea nitrogen (BUN) levels (F_3,16_ = 6.982, *p* = 0.0032). Post hoc comparisons indicated that BUN levels remained significantly lower in CoPP-treated mice compared to controls even 25 days after treatment (Fig. 3D), a sustained effect not observed with G-CSF. In contrast, glucose levels, which were reduced during the 5-day CoPP treatment (Fig. 2C), returned to baseline by day 30, with no significant differences observed between CoPP-treated and control mice (F_3,16_ = 1.081, *p* = 0.3851, Fig. 3D). No significant treatment effects were observed for other plasma parameters, including ALT (F_3,16_ = 1.062, *p* = 0.3927), ALP (F_3,16_ = 0.6423, *p* = 0.5989), total protein (F_3,16_ = 1.457, *p* = 0.2637), creatinine (F_3,16_ = 1.000, *p* = 0.4182), or creatine phosphokinase (F_3,16_ = 0.7994, *p* = 0.5121). Although aspartate transaminase (GOT/AST) activity appeared slightly elevated in both mobilized groups (G-CSF and CoPP), the differences were not statistically significant (F_3,16_ = 1.937, *p* = 0.1644, Fig. 3D).

**Fig. 4:**
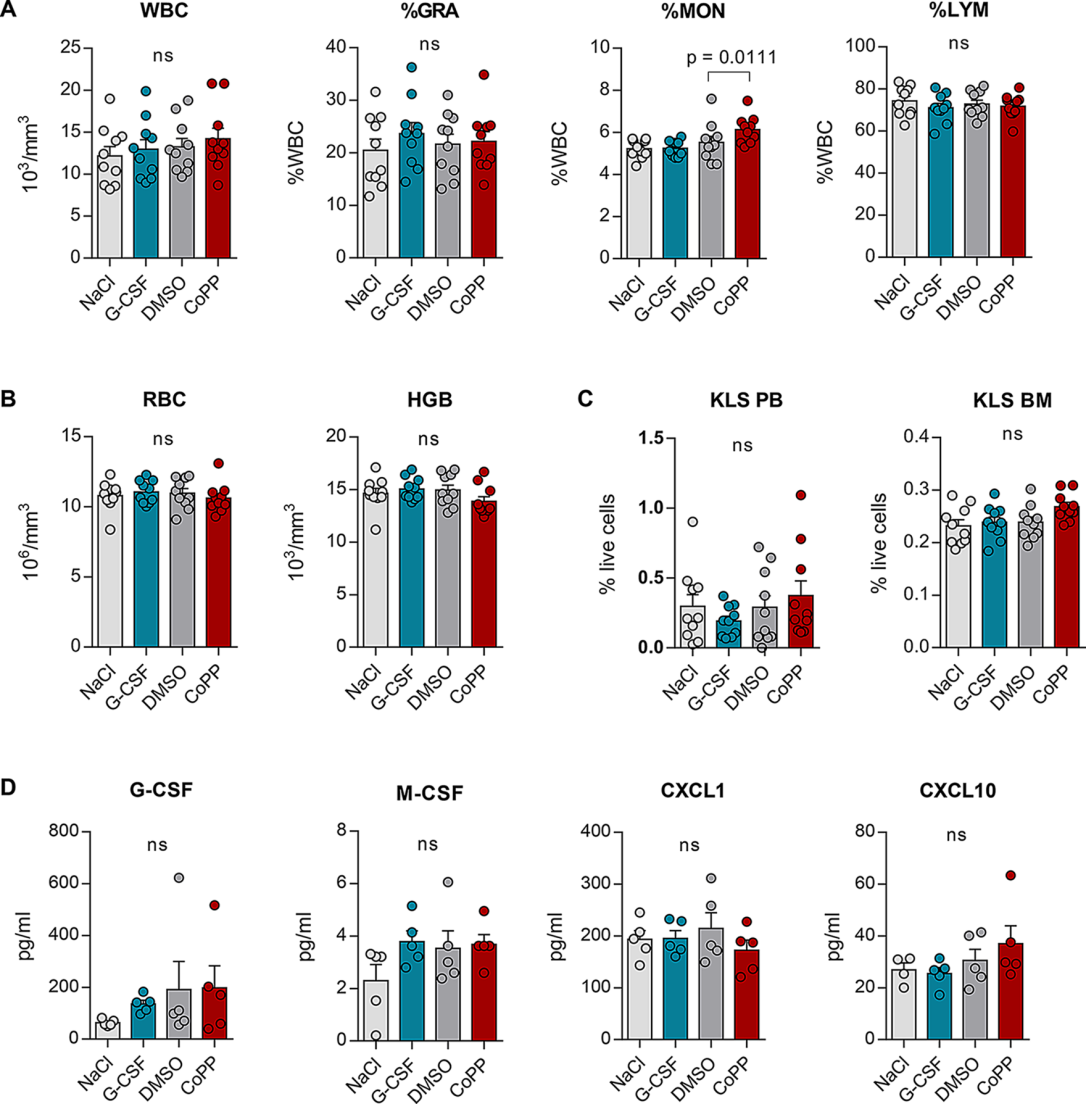
Hematological parameters and cytokine concentrations return to baseline within 25 days after the cessation of mobilization treatment. C57BL/6J mice were injected daily with 10 mg/kg CoPP, 250 µg/kg recombinant granulocyte colony-stimulating factor (G-CSF), or solvent controls (NaCl and dimethyl sulfoxide, DMSO) for 5 days. Samples were collected 25 days after the final injection. **A** Total leukocyte count in blood. White blood cell (WBC) counts and the percentages of major leukocyte populations: granulocytes (%GRA), monocytes (%MON), lymphocytes (%LYM) are comparable across all groups. **B** Red blood cell parameters (RBC – red blood cell count, HGB – hemoglobin) are similar between groups and fall within the normal range. **C** The number of c-kit⁺Lineage⁻Sca-1⁺ (KLS) cells in the blood and bone marrow is comparable between mobilized (G-CSF, CoPP) and non-mobilized (NaCl, DMSO) mice. **D** Selected cytokine and growth factor concentrations in plasma, measured by Luminex assay. Cytokine concentrations induced by CoPP return to control levels 25 days after the end of mobilization treatment. Results are shown as mean ± SEM. Statistical differences were assessed using one-way ANOVA followed by Sidak’s post-hoc test. p-values from post-hoc tests are shown in the graphs. **A**–**C** Results pooled from two independent experiments *n* = 10 mice per group. **D*** n* = 5 mice per group

As mobilization impacts hematopoietic cell populations in both the peripheral blood and bone marrow, we evaluated its long-term effects on the hematopoietic system. Blood cell counts remained similar across all groups 25 days post-treatment (Fig. 4A). The mean WBC counts ranged from to 12,190 ± 1,105 cells/µl (NaCl) to 14,230 ± 1,241 cells/µl (CoPP) and were not significantly different between the groups (F_3,36_ = 0.5649, *p* = 0.6417). The proportions of leukocyte subtypes were mostly consistent, we did not observe significant differences in the percentage of granulocytes (F_3,36_ = 0.4386, *p* = 0.7267) and lymphocytes (F_3,36_ = 0.4950, *p* = 0.6880). We only saw differences in monocytes (F_3,36_ = 4.435, *p* = 0.0094) – monocyte percentages were slightly increased in the CoPP group − 6.13 ± 0.21% compared to 5.53% ± 0.29 in the DMSO group. RBC parameters were unaffected (Fig. 4B), as neither RBC counts nor hemoglobin concentrations differed significantly between the groups (F₃,₃₆ = 0.3874, *p* = 0.7627 and F₃,₃₆ = 1.462, *p* = 0.2411, respectively).

We previously demonstrated that 5 days of treatment with CoPP, similar to G-CSF, increases the number of various subpopulations of hematopoietic stem and progenitor cells in the blood and bone marrow [11], which are included in the KLS phenotype (c-kit^+^ lineage^−^ Sca-1^+^) [31]. Importantly, the KLS numbers in the blood and bone marrow were not significantly different between the treatment groups (KLS in PB: F_3,36_ = 0.8389, *p* = 0.4815, KLS in BM: F_3,36_ = 2.648, *p* = 0.0636) indicating that the hematopoietic system had regained homeostasis 25 days after treatment (Fig. 4C).

Finally, we evaluated whether the concentrations of cytokines and growth factors induced by CoPP treatment had returned to baseline by day 30. IL5, IL6, and CCL2 levels were below detection limits, and G-CSF concentrations were similar in all groups of mice (F_3,15_ = 0.9883, *p* = 0.4248, Fig. 4D). No differences were observed in macrophage colony-stimulating factor (M-CSF), CXCL1, or CXCL10 levels between the groups (M-CSF: F_3,16_ = 1.694, *p* = 0.2084, CXCL1: F_3,16_ = 0.6610, *p* = 0.5880, CXCL10: F_3,15_ = 1.269, *p* = 0.3208).

In summary, most of the cellular and biochemical changes induced by five days of CoPP treatment resolve within one month.

## Discussion

In this study, we demonstrated that cobalt protoporphyrin IX (CoPP) effectively mobilizes hematopoietic cells from the bone marrow into peripheral blood in a dose- and time-dependent manner. Our results show that CoPP increases endogenous G-CSF and other mobilizing cytokines, leading to elevated levels of granulocytes and HSPCs in the blood. Importantly, we identified 5–10 mg/kg as the minimal effective dose range and confirmed that a 5-day treatment regimen is required for optimal mobilization. Furthermore, we evaluated the impact of a 10 mg/kg dose on the overall safety, with a focus on the long-term consequences on the mice homeostasis and potential disruptions in hematopoiesis. Since successful mobilization strategies can significantly alter the BM microenvironment and hematopoiesis [13], it was essential to assess any possible adverse effects related to these changes. While CoPP treatment caused transient changes in body weight and metabolic markers such as glucose and blood urea nitrogen (BUN), most parameters returned to baseline within 30 days.

In our previous studies, we used the dose of 10 mg/kg, which was effective in inducing G-CSF expression and mobilization of cells from the BM to the blood [11]. However, as lower doses, such as 1 and 5 mg/kg were shown to be effective in inducing biochemical changes in rodents [29, 32], we decided to investigate whether lower CoPP doses can similarly induce the mobilization. This approach is particularly relevant given that lower doses of other metalloporphyrins have been used in clinical trials. For example, in studies by Martinez et al. [33] and Kappas et al. [34], tin mesoporphyrin (SnMP) was used to prevent or treat hyperbilirubinemia in newborns. A single dose of 6 µmol/kg of birth weight was administered, corresponding to approximately 4.53 mg/kg. More recently, a phase-2 clinical trial tested RBT-1, a combination drug of tin protoporphyrin (SnPP) and iron sucrose (FeS), in patients undergoing coronary artery bypass graft or heart valve surgery. In this trial, patients received a single intravenous infusion of either high-dose RBT-1 (90 mg SnPP/240 mg FeS) or low-dose RBT-1 (45 mg SnPP/240 mg FeS) [35].

By treating mice with 1, 5, and 10 mg/kg of CoPP, we demonstrated that CoPP induces cytokine production and mobilization in a dose-dependent manner. In addition to increasing HO-1 expression dose-dependently [36, 37], CoPP has also been shown to inhibit nitric oxide synthase in the rat hypothalamus [38] and attenuate renal fibrosis in a rat model of obstructive nephropathy [36]. Interestingly, CoPP dose-dependently inhibits replication of certain viruses, for example, EqHV-8 in murine alveolar macrophage MH-S cells [39] or SARS-CoV-2 replication in the human lung epithelial Calu3 cells [40]. In our study, the lowest dose (1 mg/kg) induced G-CSF but not other cytokines detected after higher doses of CoPP, and led to a slight increase in blood granulocytes. Although this dose was insufficient for therapeutic or experimental mobilization, it’s important to note that even this low dose affected bone marrow and blood leukocytes, which may have implications for research involving tumors, inflammation, or infections.

The effect of CoPP on weight loss is well-documented, with its mechanism studied extensively in mice [32], rats [41, 42], and dogs [43]. A study by Csongradi et al. on obese melanocortin-4 receptor-deficient mice demonstrated that CoPP increases oxygen consumption, activity, and heat production, while also lowering body weight [32]. Similarly, a recent study by Rubio-Atonal et al. in rats showed that CoPP reduces food intake, water consumption, and body weight [42]. Consistent with published data, we observed a transient reduction in mouse body weight, which resolved within five days after treatment cessation. In contrast, we did not observe any weight loss in mice treated with recombinant G-CSF, so this effect is most probably not related to mobilization itself. Similarly, only in the mice treated with CoPP, we observed a transient decrease in glucose concentration. Of note, the glucose levels observed in our study were relatively high, which may be attributed to several factors, including the use of the C57BL/6 mouse strain, lack of fasting, CO₂ euthanasia [44], and the use of plasma for measurements. Notably, glucose concentrations in plasma are typically 10–15% higher than in whole blood samples [45].

We also explored whether CoPP affects other metabolic parameters, particularly liver and kidney markers, to better understand its broader physiological effects and potential toxicity. Since mobilization itself may elicit some side effects, we included a group of mice treated with G-CSF to observe the potential impacts of G-CSF and G-CSF-induced mobilization on the measured parameters. Interestingly, we observed decreased ALP activity in the plasma of mice treated with CoPP and G-CSF. A thorough literature search did not reveal any studies reporting decreased ALP activity following mobilization or treatment with G-CSF or CoPP. In contrast, several reports indicate increased ALP activity. For instance, rats treated with G-CSF at the dose of 100–300 µg/kg daily for 13 weeks exhibited increased serum ALP levels, which returned to control levels after the treatment was discontinued [46]. Additionally, a study in cancer patients found that treatment with G-CSF raised ALP levels, which returned to baseline 5–7 days after the completion of treatment [47].

Treatment with CoPP resulted in a significant decrease in BUN levels in the plasma, an effect that was evident as early as the second day of treatment and persisted for at least 25 days after discontinuation. BUN, along with creatinine, is commonly used as a marker for acute kidney injury [48]. However, in cases of kidney injury, BUN levels typically increase rather than decrease. The absence of an increase in BUN, coupled with unchanged creatinine levels, suggests that CoPP does not impair kidney function. This is further supported by the fact that creatinine is less influenced by factors such as food intake and hydration status, making it a more reliable indicator of kidney health in this context [49]. The reduction in BUN levels observed in CoPP-treated mice may instead be linked to other mechanisms. One possibility is malnutrition, as decreased food intake has been observed in CoPP-treated mice and could explain the reduction in BUN [50]. Another potential explanation for the decreased BUN levels, along with reductions in alkaline ALP activity, is the impact of CoPP on liver function. Decreased BUN levels are sometimes associated with liver dysfunction, potentially due to CoPP-induced depletion of cytochrome P450 enzymes, which are critical for hepatic metabolic processes [23]. These findings highlight the need for further studies to clarify the underlying mechanisms of BUN reduction and to assess its implications for clinical safety, particularly regarding potential effects on liver function.

In the majority of healthy human HSPC donors, transient splenomegaly is observed following G-CSF treatment [51]. However, spleen size typically returns to normal within a few days after cessation of treatment [52]. In our study, 25 days after mobilization, G-CSF-treated mice exhibited relative spleen weights comparable to those of control animals. In contrast, CoPP-treated mice showed a persistent increase in spleen weight. This observation should be considered when evaluating the clinical potential of CoPP, especially given that, although extremely rare, splenic rupture has been reported in G-CSF-mobilized patients [53]. G-CSF-induced splenomegaly is thought to result from enhanced splenic erythropoiesis [52], particularly under conditions of systemic inflammation [54]. It is possible that cytokines induced by CoPP may prolong the time required for the spleen size to return to baseline.

In addition to assessing toxicity, understanding the dynamics of granulocyte production and lifespan is crucial for interpreting the effects of CoPP treatment. Granulocytes, including neutrophils, have a relatively short lifespan in circulation, typically ranging from 6 to 18 h [55, 56]. Their production can be rapidly increased in response to G-CSF stimulation [57]. Given that recombinant G-CSF is commonly administered for 5 days in clinical settings for neutropenia treatment [58] and HSCs donor mobilization [28], we adopted this 5-day treatment regimen for the majority of our experiments. Consistent with this approach, our experimental data demonstrated that a 5-day treatment period resulted in the most efficient mobilization of white blood cells, compared to shorter treatment durations. For long-term effects, we selected a 30-day follow-up timepoint, as it aligns with the standard first follow-up period for stem cell donors after blood donation, during which the majority of blood parameters are expected to return to baseline levels, as observed in clinical studies [28, 59]. By examining hematological parameters in mice treated with CoPP for 5 days and then 25 days after the treatment ended, we demonstrated that the effects of CoPP on leukocyte counts, red blood cell parameters, and HSPCs in the BM are transient. Given the proposed therapeutic uses of CoPP [26, 27, 60], understanding its impact on the hematopoietic system and ensuring it does not compromise long-term hematopoiesis is essential.

In summary, this study defined the optimal dose and duration of CoPP treatment to effectively induce the mobilization of granulocytes into peripheral blood in mice. We also demonstrated that most (though not all) of the changes elicited by CoPP resolve within 30 days. These findings suggest that CoPP could potentially be used as a drug for inducing granulocyte mobilization. However, further studies are needed to elucidate the mechanisms by which CoPP affects parameters such as BUN and ALP. Additionally, if CoPP is considered for potential therapeutic use beyond mobilization, its effects on the hematopoietic system must be carefully evaluated. Although our study has some limitations, including the lack of human trials with CoPP and the potential for species-specific variability, there are several key points worth noting. While CoPP has not yet been tested in clinical trials, similar effects have been observed in other species, such as rats and dogs, suggesting that its biological activity may be consistent across different models. Additionally, other metalloporphyrins, such as tin mesoporphyrin (SnMP), have demonstrated similar mechanisms of action in both humans and mice. These findings underscore the potential of CoPP as a therapeutic agent, though further studies are necessary to fully establish its efficacy and safety in human populations.

## Electronic supplementary material

Below is the link to the electronic supplementary material.

Supplementary Material 1

## Data Availability

The datasets generated during and/or analyzed during the current study are available from the corresponding author upon reasonable request. Upon publication of all project results, the data will be made publicly accessible through the RODBUK Cracow Open Research Data Repository (https://rodbuk.pl/).

## Abbreviations

%GRA: Percentage of granulocytes
%LYM: Percentage of lymphocytes
%MON: Percentage of monocytes
ALP: Alkaline phosphatase
AST: Aspartate transaminase
b.w.: Body weight
BM: Bone marrow
BUN: Blood urea nitrogen
CCL2: C-C Motif Chemokine Ligand 2
CO: Carbon monoxide
CoPP: Cobalt protoporphyrin IX
Cre: Creatinine
CXCL: C-X-C Motif Chemokine Ligand
DMSO: Dimethyl sulfoxide
FBS: Fetal bovine serum
FeS: Iron sucrose
G-CSF: Granulocyte colony-stimulating factor
Glu: Glucose
GPT/ALT: Alanine transaminase
HCT: Hematocrit
HO-1: Heme oxygenase-1
HSCs: Hematopoietic stem cells
HSPCs: Hematopoietic stem and progenitor cells
IL: Interleukin
KLS: c-kit^+^ lineage^−^ Sca-1^+^
LPS: Lipopolysaccharide
M-CSF: Macrophage Colony-Stimulating Factor
PB: Peripheral blood
SnMP: Tin mesoporphyrin
T-Pro: Total protein
WBC: White blood cells

## References

[1] Tenhunen R, Marver HS, Schmid R. The enzymatic conversion of Heme to bilirubin by microsomal Heme Oxygenase. Proc Natl Acad Sci U S A. 1968;61:748–55. 10.1073/pnas.61.2.748 PMC225223 4386763

[2] Tenhunen R, Ross ME, Marver HS, Schmid R. Reduced nicotinamide adenine dinucleotide phosphate dependent biliverdin reductase. Partial purification and characterization. Biochemistry. 1970;9:298–303. 4391687 10.1021/bi00804a016

[3] Blancou P, Tardif V, Simon T, Rémy S, Carreño L, Kalergis A, et al. Immunoregulatory properties of Heme oxygenase-1. In: Cuturi MC, Anegon I, editors. Suppression and regulation of immune responses: methods and protocols. Totowa, NJ: Humana; 2011. pp. 247–68. 10.1007/978-1-60761-869-0_18 20941616

[4] Otterbein LE, Bach FH, Alam J, Soares M, Tao Lu H, Wysk M, et al. Carbon monoxide has anti-inflammatory effects involving the mitogen-activated protein kinase pathway. Nat Med. 2000;6:422–8. 10742149 10.1038/74680

[5] Lee TS, Chau LY. Heme oxygenase-1 mediates the anti-inflammatory effect of interleukin-10 in mice. Nat Med. 2002;8:240–6. 11875494 10.1038/nm0302-240

[6] Loboda A, Jazwa A, Grochot-Przeczek A, Rutkowski AJ, Cisowski J, Agarwal A, et al. Heme oxygenase-1 and the vascular bed: from molecular mechanisms to therapeutic opportunities. Antioxid Redox Signal. 2008;10:1767–812. 18576916 10.1089/ars.2008.2043

[7] Ryter SW, Alam J, Choi AMK. Heme oxygenase-1/carbon monoxide: from basic science to therapeutic applications. Physiol Rev. 2006;86:583–650. 16601269 10.1152/physrev.00011.2005

[8] Blumenthal SB, Kiemer AK, Tiegs G, Seyfried S, Höltje M, Brandt B, et al. Metalloporphyrins inactivate caspase-3 and– 8. FASEB J. 2005;19:1272–9. 16051694 10.1096/fj.04-3259com

[9] Lin HY, Shen SC, Lin CW, Wu MS, Chen YC. Cobalt protoporphyrin Inhibition of lipopolysaccharide or Lipoteichoic acid-induced nitric oxide production via blocking c-Jun N-terminal kinase activation and nitric oxide enzyme activity. Chem Biol Interact. 2009;180:202–10. 19497418 10.1016/j.cbi.2009.01.004

[10] Lin HY, Tsai CH, Lin C, Yeh WL, Tsai CF, Chang PC, et al. Cobalt protoporphyrin upregulates Cyclooxygenase-2 expression through a Heme Oxygenase-Independent mechanism. Mol Neurobiol. 2016;53(7):4497–508. 26255181 10.1007/s12035-015-9376-y

[11] Szade A, Szade K, Nowak WN, Bukowska-Strakova K, Muchova L, Gońka M, et al. Cobalt protoporphyrin IX increases endogenous G-CSF and mobilizes HSC and granulocytes to the blood. EMBO Mol Med. 2019;11:e09571. 31709729 10.15252/emmm.201809571 PMC6895613

[12] Weiss L, Geduldig U. Barrier cells: stromal regulation of hematopoiesis and blood cell release in normal and stressed murine bone marrow. Blood. 1991;78:975–90. 1868254

[13] Lapid K, Glait-Santar C, Gur-Cohen S, Canaani J, Kollet O, Lapidot T. In: StemBook, editor. Egress and mobilization of hematopoietic stem and progenitor cells: a dynamic multi-facet process. Cambridge (MA): Harvard Stem Cell Institute; 2008. 23658994

[14] Blayney DW, Schwartzberg L. Chemotherapy-induced neutropenia and emerging agents for prevention and treatment: A review. Cancer Treat Rev. 2022;109:102427. 35785754 10.1016/j.ctrv.2022.102427

[15] Luo C, Wang L, Wu G, Huang X, Zhang Y, Ma Y, et al. Comparison of the efficacy of hematopoietic stem cell mobilization regimens: a systematic review and network meta-analysis of preclinical studies. Stem Cell Res Ther. 2021;12:310. 34051862 10.1186/s13287-021-02379-6 PMC8164253

[16] Menendez-Gonzalez JB, Hoggatt J. Hematopoietic stem cell mobilization: current collection approaches, stem cell heterogeneity, and a proposed new method for stem cell transplant conditioning. Stem Cell Rev Rep. 2021;17:1939–53. 34661830 10.1007/s12015-021-10272-1 PMC8663585

[17] Bendall LJ, Bradstock KF. G-CSF: from granulopoietic stimulant to bone marrow stem cell mobilizing agent. Cytokine Growth Factor Rev. 2014;25:355–67. 25131807 10.1016/j.cytogfr.2014.07.011

[18] To LB, Levesque JP, Herbert KE. How I treat patients who mobilize hematopoietic stem cells poorly. Blood. 2011;118:4530–40. 21832280 10.1182/blood-2011-06-318220

[19] Hoggatt J, Pelus LM. New G-CSF agonists for neutropenia therapy. Expert Opin Investig Drugs. 2014;23:21–35. 24073859 10.1517/13543784.2013.838558

[20] Kuang P, Lin T, Chen X, Yang Y, Ji J, Dong T, et al. Plerixafor as a preemptive or salvage therapy for healthy donors with poor mobilization of hematopoietic stem cells. Bone Marrow Transpl. 2022;57:1737–9. 10.1038/s41409-022-01789-1 PMC9630128 36076012

[21] Gazendam RP, van de Geer A, van Hamme JL, Tool ATJ, van Rees DJ, Aarts CEM, et al. Impaired killing of Candida albicans by granulocytes mobilized for transfusion purposes: a role for granule components. Haematologica. 2016;101:587–96. 26802050 10.3324/haematol.2015.136630 PMC5004374

[22] Müller AMS, Linderman JA, Florek M, Miklos D, Shizuru JA. Allogeneic T cells impair engraftment and hematopoiesis after stem cell transplantation. Proc. Natl. Acad. Sci. 2010;107:14721–6. 10.1073/pnas.1009220107 PMC2930440 20679222

[23] Muhoberac BB, Hanew T, Halter S, Schenker S. A model of cytochrome P-450-centered hepatic dysfunction in drug metabolism induced by cobalt-protoporphyrin administration. Biochem Pharmacol. 1989;38:4103–13. 2512932 10.1016/0006-2952(89)90692-8

[24] Nebert DW, Russell DW. Clinical importance of the cytochromes P450. The Lancet. 2002;360:1155–62. 12387968 10.1016/S0140-6736(02)11203-7

[25] Deodhar M, Al Rihani SB, Arwood MJ, Darakjian L, Dow P, Turgeon J, et al. Mechanisms of CYP450 inhibition: Understanding Drug-Drug interactions due to Mechanism-Based Inhibition in clinical practice. Pharmaceutics. 2020;12:846. 32899642 10.3390/pharmaceutics12090846 PMC7557591

[26] Li J, Wu B, Teng D, Sun X, Li J, Li J, et al. Cobalt-protoporphyrin enhances Heme Oxygenase 1 expression and attenuates liver ischemia/reperfusion injury by inhibiting apoptosis. Mol Med Rep. 2018;17:4567–72. 29328470 10.3892/mmr.2018.8384

[27] Fang PH, Lai YY, Chen CL, Wang HY, Chang YN, Lin YC, et al. Cobalt protoporphyrin promotes human keratinocyte migration under hyperglycemic conditions. Mol Med. 2022;28:71. 35739477 10.1186/s10020-022-00499-0 PMC9219158

[28] Hölig K, Kramer M, Kroschinsky F, Bornhäuser M, Mengling T, Schmidt AH, et al. Safety and efficacy of hematopoietic stem cell collection from mobilized peripheral blood in unrelated volunteers: 12 years of single-center experience in 3928 donors. Blood. 2009;114:3757–63. 19666868 10.1182/blood-2009-04-218651

[29] Peng PH, Chao HM, Juan SH, Chen CF, Liu JH, Ko ML. Pharmacological preconditioning by low dose Cobalt protoporphyrin induces Heme Oxygenase-1 overexpression and alleviates retinal Ischemia-Reperfusion injury in rats. Curr Eye Res. 2011;36:238–46. 21275512 10.3109/02713683.2010.539760

[30] Besenzon F, Dedja A, Vadori M, Bosio E, Seveso M, Tognato Ei in. In vitro and in vivo Immunomodulatory effects of Cobalt protoporphyrin administered in combination with immunosuppressive drugs. Transpl Immunol. 2010;24:1–8. 20713156 10.1016/j.trim.2010.08.002

[31] Lin KK, Goodell MA. Detection of hematopoietic stem cells by flow cytometry. In: Darzynkiewicz Z, Holden E, Orfao A, Telford W, Wlodkowic D, editors. Methods in cell biology. Academic; 2011. pp. 21–30. 10.1016/B978-0-12-385493-3.00002-4 21722798

[32] Csongradi E, Docarmo JM, Dubinion JH, Vera T, Stec DE. Chronic HO-1 induction with Cobalt protoporphyrin (CoPP) treatment increases oxygen consumption, activity, heat production and lowers body weight in obese melanocortin-4 receptor-deficient mice. Int J Obes 2005. 2012;36:244–53. 10.1038/ijo.2011.78 PMC3139690 21467998

[33] Martinez JC, Garcia HO, Otheguy LE, Drummond GS, Kappas A. Control of severe hyperbilirubinemia in full-term newborns with the inhibitor of bilirubin production Sn-mesoporphyrin. Pediatrics. 1999;103:1–5. 9917431 10.1542/peds.103.1.1

[34] Kappas A, Drummond GS, Valaes T. A single dose of Sn-mesoporphyrin prevents development of severe hyperbilirubinemia in glucose-6-phosphate dehydrogenase-deficient newborns. Pediatrics. 2001;108:25–30. 11433050 10.1542/peds.108.1.25

[35] Lamy A, Chertow GM, Jessen M, Collar A, Brown CD, Mack CA et al. Effects of RBT-1 on preconditioning response biomarkers in patients undergoing coronary artery bypass graft or heart valve surgery: a multicentre, double-blind, randomised, placebo-controlled phase 2 trial. eClinicalMedicine. 2024;68. 10.1016/j.eclinm.2023.102364 PMC10994969 38586479

[36] Iwai T, Kitamoto K, Teramoto K, Machida Y, Tamada S, Yukimura T, et al. Cobalt protoporphyrin attenuates rat obstructive nephropathy: role of cellular infiltration. Urology. 2008;72:432–8. 18313104 10.1016/j.urology.2007.11.123

[37] Yashima Y, Okamoto K, Sakai E, Iwatake M, Fukuma Y, Nishishita K, et al. Cobalt protoporphyrin represses osteoclastogenesis through blocking multiple signaling pathways. Biometals. 2015;28:725–32. 25981584 10.1007/s10534-015-9861-9

[38] Li M, Vizzard MA, Jaworski DM, Galbraith RA. The weight loss elicited by Cobalt protoporphyrin is related to decreased activity of nitric oxide synthase in the hypothalamus. J Appl Physiol Bethesda Md 1985. 2006;100:1983–91. 10.1152/japplphysiol.01169.2005 16469935

[39] Li L, Hu X, Li S, Li Y, Zhao S, Shen F, et al. Cobalt protoporphyrin blocks EqHV-8 infection via IFN-α/β production. Animals. 2023;13:2690. 37684954 10.3390/ani13172690 PMC10487175

[40] Zhang S, Wang J, Wang L, Aliyari S, Cheng G. SARS-CoV-2 virus NSP14 impairs NRF2/HMOX1 activation by targeting Sirtuin 1. Cell Mol Immunol. 2022;19:872–82. 35732914 10.1038/s41423-022-00887-w PMC9217730

[41] Choi SJ, Meeran K, O’Shea D, Lambert PD, Bloom SR. Weight loss in rats treated with intracerebroventricular Cobalt protoporphyrin is not specific to the neuropeptide Y system. Brain Res. 1996;729:223–7. 8876991 10.1016/s0006-8993(96)00423-4

[42] Rubio-Atonal LF, Serrano-García N, Limón-Pacheco JH, Pedraza-Chaverri J, Orozco-Ibarra M. Cobalt protoporphyrin decreases food intake, body weight, and the number of neurons in the nucleus accumbens in female rats. Brain Res. 2021;1758:147337. 33548272 10.1016/j.brainres.2021.147337

[43] Galbraith RA, Kappas A. Cobalt protoporphyrin regulates body weight in beagle dogs: induction of weight loss in normal animals of stable adult weight. Pharmacology. 2008;43:96–105. 10.1159/000138834 1775515

[44] Fitzhugh DC, Parmer A, Shelton LJ, Sheets JT. A comparative analysis of carbon dioxide displacement rates for euthanasia of the ferret. Lab Anim. 2008;37:81–6. 10.1038/laban0208-81 PMC7091634 18216799

[45] Kotwal N, Pandit A. Variability of capillary blood glucose monitoring measured on home glucose monitoring devices. Indian J Endocrinol Metab. 2012;16:S248–51. 23565391 10.4103/2230-8210.104052 PMC3603039

[46] Kato Y, Yamamoto M, Ikegami J, Okumura S, Hara T, Shuto K. A possible mechanism of increase in serum alkaline phosphatase activity in rats given granulocyte Colony-Stimulating factor. Exp Anim. 1996;45:23–32. 8689577 10.1538/expanim.45.23

[47] Fukumasu H, Fukumasu Y, Ogita S. Elevation in plasma alkaline phosphatase level during rhG-CSF administration: granulocytopenic patients with gynecologic cancers treated with Cancer chemotherapy. Gynecol Obstet Invest. 1998;45:99–104. 9517801 10.1159/000009934

[48] Edelstein CL. Biomarkers of acute kidney injury. Adv. Chronic Kidney Dis. 2008;15:222–34. 18565474 10.1053/j.ackd.2008.04.003 PMC3287955

[49] Washington IM, Van Hoosier G. Clinical biochemistry and hematology. In: Suckow MA, Stevens KA, Wilson RP, editors. The laboratory rabbit, guinea pig, hamster, and other rodents. Boston: Academic; 2012. pp. 57–116.

[50] Hosten AO. BUN and creatinine. In: Walker HK, Hall WD, Hurst JW, editors. Clinical methods: the history, physical, and laboratory examinations. Boston: Butterworths; 1990. 21250045

[51] Platzbecker U, Prange-Krex G, Bornhäuser M, Koch R, Soucek S, Aikele P, et al. Spleen enlargement in healthy donors during G–CSF mobilization of PBPCs. Transfus (Paris). 2001;41:184–9. 10.1046/j.1537-2995.2001.41020184.x 11239220

[52] Stroncek D, Shawker T, Follmann D, Leitman SF. G-CSF-induced spleen size changes in peripheral blood progenitor cell donors. Transfus (Paris). 2003;43:609–13. 10.1046/j.1537-2995.2003.00384.x 12702182

[53] Benguerfi S, Thepault F, Lena H, Ricordel C. Spontaneous Splenic rupture as a rare complication of G-CSF injection. BMJ Case Rep. 2018;2018:bcr2017222561. 29330272 10.1136/bcr-2017-222561 PMC5780587

[54] Jing W, Guo X, Qin F, Li Y, Wang G, Bi Y et al. G-CSF shifts erythropoiesis from bone marrow into spleen in the setting of systemic inflammation. Life Sci Alliance. 2021;4. 10.26508/lsa.202000737 PMC7723243 33234677

[55] Strydom N, Rankin SM. Regulation of Circulating neutrophil numbers under homeostasis and in disease. J Innate Immun. 2013;5:304–14. 23571274 10.1159/000350282 PMC6741587

[56] Boivin G, Faget J, Ancey PB, Gkasti A, Mussard J, Engblom C, et al. Durable and controlled depletion of neutrophils in mice. Nat Commun. 2020;11:2762. 32488020 10.1038/s41467-020-16596-9 PMC7265525

[57] Panopoulos AD, Watowich SS. Granulocyte colony-stimulating factor: molecular mechanisms of action during steady state and ‘emergency’ hematopoiesis. Cytokine. 2008;42:277–88. 18400509 10.1016/j.cyto.2008.03.002 PMC2852428

[58] Clemons M, Fergusson D, Simos D, Mates M, Robinson A, Califaretti N, et al. A multicentre, randomised trial comparing schedules of G-CSF (filgrastim) administration for primary prophylaxis of chemotherapy-induced febrile neutropenia in early stage breast cancer. Ann Oncol. 2020;31:951–7. 32325257 10.1016/j.annonc.2020.04.005

[59] Coluccia P, Crovetti G, Del Fante C, Dallavalle FM, Laszlò D, Ferremi P, et al. Screening of related donors and peripheral blood stem cell collection practices at different Italian apheresis centres. Blood Transfus. 2012;10:440–7. 22871823 10.2450/2012.0140-11 PMC3496225

[60] Shan Y, Lambrecht RW, Donohue SE, Bonkovsky HL. Role of Bach1 and Nrf2 in up-regulation of the Heme oxygenase-1 gene by Cobalt protoporphyrin. FASEB J Off Publ Fed Am Soc Exp Biol. 2006;20:2651–3. 10.1096/fj.06-6346fje 17065227

