# Supplementary material for: Cobalt protoporphyrin IX induces transient, dose- and time-dependent granulocyte mobilization with mild metabolic effects in mice
Source: Pharmacol Rep. 2025 Jul 9;77(5):1295–308. doi: 10.1007/s43440-025-00751-4 (PMC12443917; doi:10.1007/s43440-025-00751-4)
Supplement: Supplementary file 1 — Supplementary Material 1 [file 43440_2025_751_MOESM1_ESM.pdf]

Suppl. Fig. 1

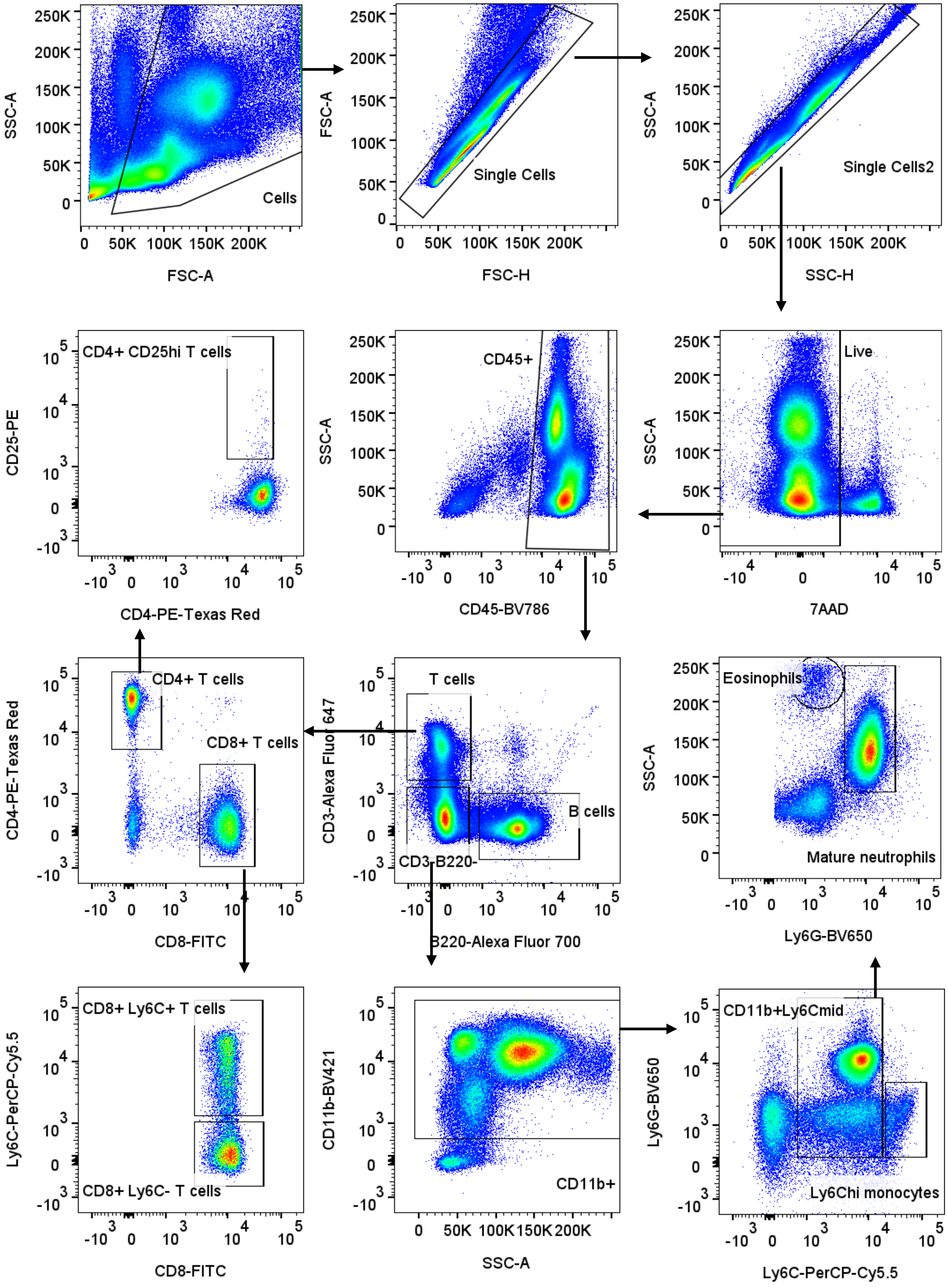

Gating strategy for the analysis of peripheral blood leukocyte subpopulations.

Suppl. Fig. 2

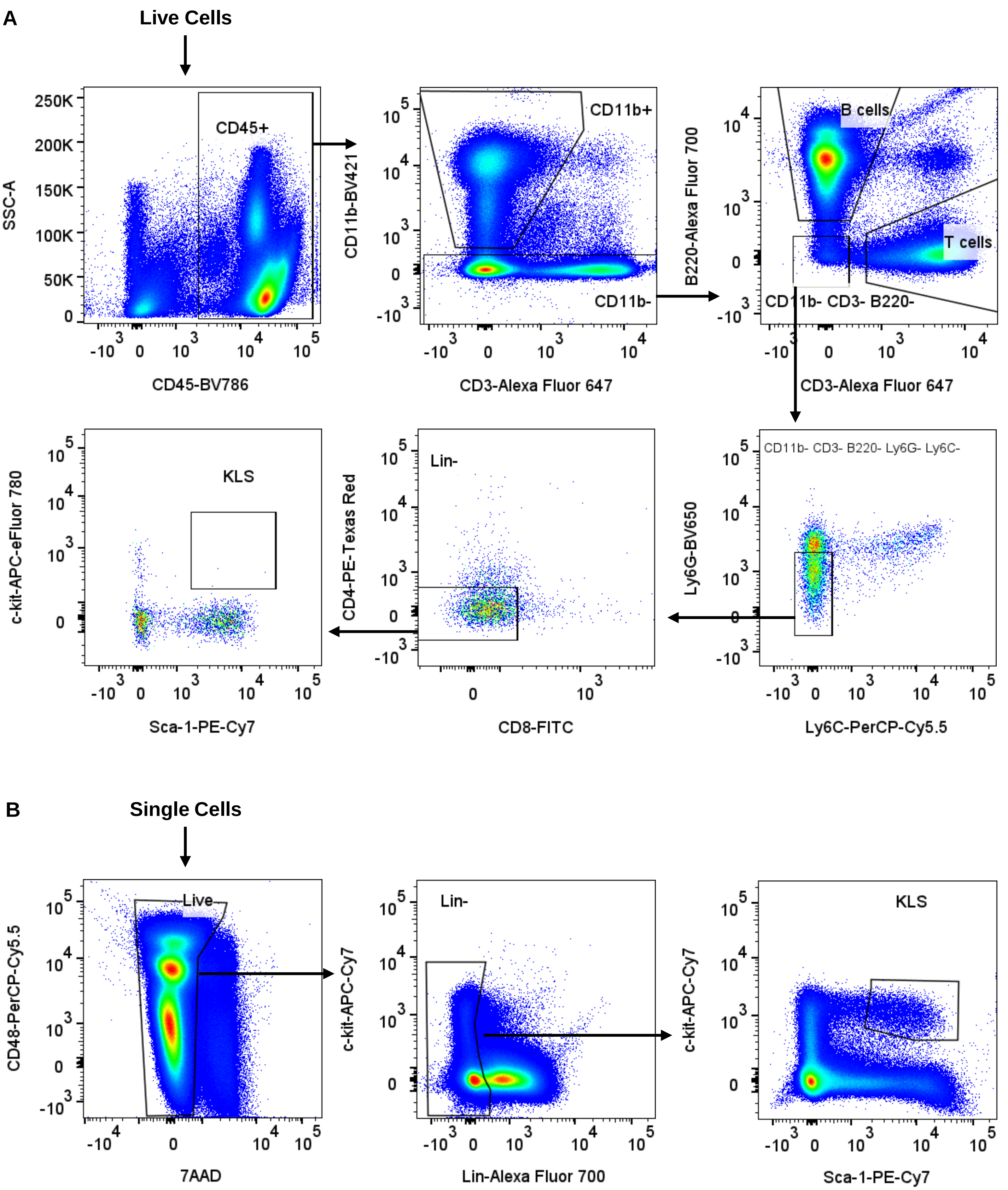

Gating strategy for the analysis of c-Kit^{+} Lin^{-} Sca-1^{+} (KLS) cells in peripheral blood (A) and bone marrow (B).

Suppl. Fig. 3

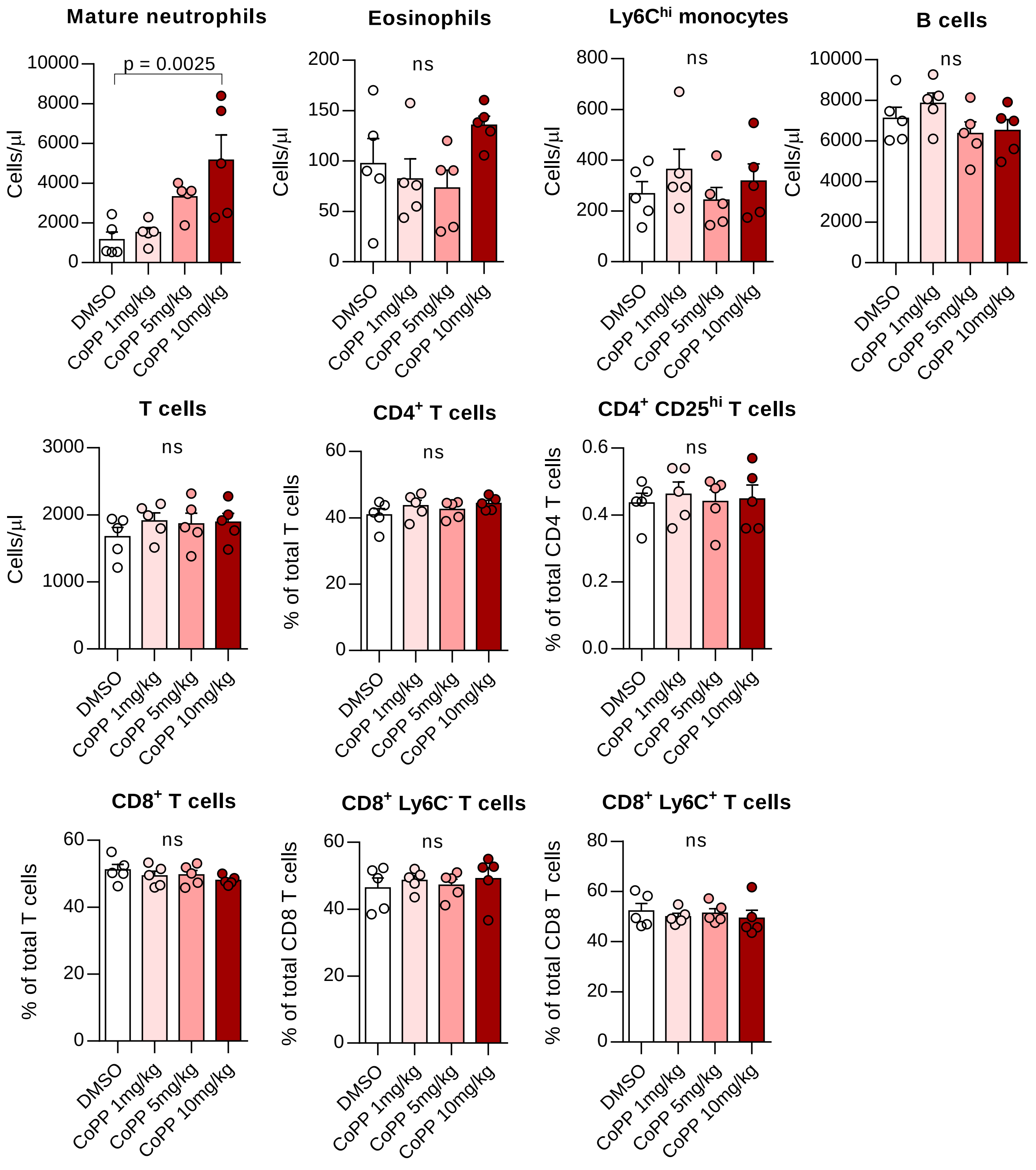

Flow cytometry analysis of leukocyte subpopulations in the peripheral blood (PB) of C57BL/6J mice following a single injection of cobalt protoporphyrin IX (CoPP) or solvent control (DMSO). Blood samples were collected 6 hours post-injection. Data are presented as mean ± SEM. Statistical significance was assessed using one-way ANOVA followed by Dunnett's post-hoc test. p-values from post-hoc comparisons are shown on the graphs. n = 5 mice per group
